# Theories of the Production of Males and Females

**Published:** 1855-04

**Authors:** Silas Hubbard

**Affiliations:** Buffalo


### ART. II.— *Theories of the Production of Males and Females.*

By Silas Hubbard, M. D.

The causes which decide the sex of the fœtus are acknowledged in the above title (Theories) to be still more matters of speculation than of knowledge. But we have some very respectable authority encouraging us to investigate this subject, and to "prove all things and hold fast that which is good." For this reason, and because almost all physiological discoveries were first theories and then science, I hope I may be pardoned for offering for the investigation of such of my medical brethren as may feel an interest therein, a few suggestions on the subject above indicated. To further show that it is worthy of attention I will also quote the opinions of several authors, some of whom are very respectable.

To produce either sex the Egyptians and Indians depended on the state of the heavens, or on the position of the constellations at the moment of fecundation; the Greeks and all the people of the ancient world used to rely upon the phases of the moon, &c.

The ancient agriculturists were convinced, and many country people still think, that if the north wind prevails, that if the season be dry and cool rather than warm and moist, when goats, sheep and cows are admitted to the males, there will be fewer females produced than under the influence of a contrary state of the atmosphere.

The older philosophers, as Hippocrates and Aristotle, believed that the right testicle and ovary furnish the rudiments of males, and the same organs on the left side, those of females: some of the old writers, *de Re Rusticâ*, assert, indeed, that such was the result of their experiments with the ram. These statements gave rise to a pretended "art of procreating the sexes at pleasure," which has even been seriously revived in our own time. Milot boldly affirmed that males are produced by the right ovarium, and females by the left; asserting that he could so manage the position of the woman during copulation, that she should certainly have a boy or girl, as might have been determined upon; but I need not say these theories have been fully and clearly proved to be erroneous.

Mr. Babbage and several other authors have collected extensive statistics showing that the proportion of male children is greater in cases of legitimate than of illegitimate births.

It has been attempted to show that the corporeal vigor of the parents has much to do with the future sex. M. Giron instituted a series of experiments on different animals, but especially on sheep, to discover whether a greater number of male or female lambs may not be produced at the will of the agriculturist. The plan adopted to insure this result, was to employ very young rams, in that division of the flock whence it was desired to obtain females; and strong and vigorous rams, of four or five years of age, in that from which males were to be produced. The result would seem to show, that the younger rams begat females in greater proportion, and the older, males. M. Giron asserts, that females commonly predominate among animals that live in a state of "polygamy." These and other facts have seemed to some to prove, that in the act of generation, it is, as a general rule, the stronger individual that regulates the sex of the progeny. M. Moreau has arrived at this conclusion as the result of long observation. He is of opinion that, to a certain extent, a boy or girl may be begotten at will by strengthening or weakening the father or mother, previous to the act of generation; and he states, that by acting on this rule he has seen in numerous instances his advice followed by the desired result.

From the researches of Hofacer and Sadler, it would seem that, as a general rule, when the mother is older than the father, fewer boys are born than girls, and the same is observed where they are of equal age; but the greater the excess of age on the part of the father, the greater will be the ratio of boys born.

Recent researches by Dr. Emerson, have led him to the conclusion, that the extensive prevalence of every severe zymotic disease, and, indeed, any occurrence which directly or indirectly exerts a decidedly depressing effect upon a community, will be indicated in the record of births, by a conspicuous reduction in the proportion of males. The ordinary average excess of male births was found, by former calculations, to be, in Philadelphia, about 7 per cent.; and during the cholera months of August and September, 1832, the diminution of male conceptions was at the rate of more than 17 per cent.; and a similar diminution occurred in Paris, and other places, during the existence of the same malady. Dr. Emerson further says, "Investigations into the comparative proportions of the sexes born in city and country populations, manifest the existence of a greater male excess in rural districts," &c.

It is well known to those who understand the history of the honey-bee, that they know how to convert their larvæ into males and females and mules, or working bees, as their knowledge or instinct or necessities may require; and now I ask may not man possibly, with his boasted reason, likewise yet learn, to a very considerable extent, how to propagate the sexes as he may will?

Velpeau says, "in a flock of sheep, those that are first covered produce fewer males than those that come immediately after them, and these many more than the last moiety; for the ram does not appear to enjoy his whole prolific energy until after a certain number of copulations, and afterward he becomes exhausted, graduallp losing his strength."

Velpeau, and many other respectable authors whose names I have not mentioned, express the strong hope that we will yet discover how to control the causes which operate to modify the sex.

Now as I shall have occasion in my discourse to refer to some of the foregoing statements of authors, I will here give some of my own observations, to which I shall also afterward refer. I have observed that those women who are strong and exercise the most on foot or on horseback, as a class, give birth to a greater ratio of males than those women who are sickly, or feeble, or sedentary in their habits or employments. Any one can demonstrate this by making a list of each class of his acquaintances and reckoning up the boys and girls of each, and then averaging them. Hence a physician may, with some degree of success, prescribe to that woman who desires a son that she should daily take considerable exercise on foot or on horseback, till she is convinced pregnancy has taken place, for as soon as it has occurred I believe the destiny of the embryo as to its sex is fixed; and, if she wishes a daughter, advise her to engage in only sedentary employments. It may be possible that these respective causes may operate up to fourteen weeks of pregnancy, because up to that period the particular sex cannot be distinguished. Here let me say, *en passant*, that it would be interesting if physiologists would also demonstrate at what age of the embryo of various animals the particular sex seems to begin to be superimposed.

In the September No., 1850, of this Journal, I advanced the theory that males are conceived shortly before the time of the courses, and females shortly after; and now I propose to treat of this theory more fully, and to give my reasons therefor. My theory is that there is generally a periodical development and maturation of an ovum near the time of the courses, and that said maturation usually bears the same relation to the time of the courses in all women, and thus they are ordinarily more susceptible of impregnation shortly before and shortly after menstruation: and also that this susceptibility is nearly equal at both these times. My theory further is that the same ovum, if fecundated shortly before the courses, will generally grow to be a male, while if its fecundation is deferred till after the courses, it will generally grow to be a female. If the maturition of the ovum bears a particular relation to the time of the courses, then it must be acknowledged that its condition must be very different before to what it is after the menses; indeed, a great difference can be seen, at different ages of its ripeness, with the microscope. It necessarily follows, then, that if fecundation occurs in one of these cases, the embryo will grow up to be a very different being to what it would in the other: that is to say, the ovum which grows to be a male is fecundated as soon as it is sufficiently mature to be impregnated, or while it is quite recent; but if its fecundation is postponed to a particular period later, it grows to be a female. I will not say that this rule relative to the maturition of an ovum near the time of the courses will always hold true, because rarely it is matured and fecundated during the intermenstrual period without any reference to the time of the menses, which is an exception to the general rule.

Granting that the above theories are correct, still there may possibly be a difference of ability of males to procreate one sex or the other, *i. e.*, the sperm of one male though it might have sufficient strength to fecundate a germ within the uterus, or very nearly within it, might not be sufficient to impregnate one so far distant as the ovary or fimbriated extremity of the fallopian tube, while another could, and thus would result a difference of power of procreating males.

If pregnancy occurs shortly before the time of the courses, the menses first due generally appear, though not always; but if it occurs shortly after, then they very rarely appear again till after delivery.

The ova may be fecundated while in the ovaria or fallopian tubes, or uterus. The latter, I expect, become females, and the former males. Hence extra-uterine conceptions are more likely to be males. (See my article on "Cause of Extra-uterine Conceptions," page 215, vol. vii. of this Journal.)

Founding on the principle that the male embryo is sooner developed than the female, Aristotle says, as well as Hippocrates and many other ancient authors, that the woman quickens earlier with a boy and later with a girl. I have heard women who had had several children of both sexes, say this was their experience, and also declare that their boys were born a week or several days sooner from the last appearance of their courses than their girls; and one woman with a large family of both sexes, on whose word I could rely, declared that she carried her boys eight months and her girls nine months from the last appearance of the menses. These statements and several corroborative cases, were what first led me to suppose that boys are conceived shortly before the courses, for the consequence is evident that in such cases they would quicken and be born earlier after the last appearance of the menses than if the germ had been fecundated after menstruation.

The probability that those children who are united or grown together, were conceived at the same time, and under the same circumstances, and thus are of the same sex, also affords some confirmation of the foregoing theories. (See my article on "Blending of Twins," page 546, vol. vii., of this Journal.)

As we would naturally expect, twins are generally of the same sex, and I expect they are always of the same sex when they are inclosed in the same membranes. I should as much expect that they are of the same sex when heir secundines are blended as when they are blended themselves. Those children who are united and also those who were inclosed within the same membranes, are probably offshoots from the same ovum, analagous to a double yolk hen's egg, or a two kernel almond; in other words, there is good ground to believe that such a multiplication by subdivision may take place at the earliest period of embryonic life, at which the germ is nothing else than a mass of cells, wherein no distinction of parts has as yet manifested itself; and that the production of two complete individuals, only held together by a connecting band, may arise from some cause which determines the subdivision of the germinal mass, at the period when its grade of development corresponds with that of the Hydra or Planaria. And this view of the case is confirmed by the fact that there is a higher degree of regenerating power during embryonic life, infancy, and childhood, as compared with that which remains after the development of the fabric has been completed. If these statements are correct, then it is certain that such children were conceived at the same time and under the same circumstances.

Probably more ova which would grow to be females are fecundated than the other, because they are nearer in reach of the semen; but they are also oftener cast off because they do not get so early a start as to be so likely to make a fastening, so that the chances of male births are rather greater than female. Extensive and reliable statistics show that there are twenty-one males born to twenty females; but the preponderance of male deaths in childhood, reduces the number of both sexes to about an equality in adult life.

If the Jewish nation could be depended on as "stiffnecked" law abiders, they would furnish great proof or disproof of the foregoing statements, for they have a scriptural law which they profess to obey, that no man shall touch a woman till eight days after her courses; but I have no faith that they generally obey this law, or any other law to restrain their lusts that they can secretly violate. If they did obey the said law, then according to my theory, they would have a great proportion of males.

Hermaphrodites are probably begotten during the courses, or at a midway time of ripeness of the ova, or at least in such case the product, if a female, will appear masculine, or if a male will appear feminine.

Those women who are subject to the irregularities of having their courses return too frequently, or to continue more or less for too long a period, are, during such irregularities, more likely to conceive boys; because unfecundated ova within the uterus, are more likely to be washed away by the menstrua before they could acquire attaching power; while the ova which grow to be males acquire attaching power before they arrive within the uterus. I have several cases in favor of these statements.

I would advise every physician not to be discouraged and give up these theories entirely because a boy happened to be conceived immediately after the courses, as there are exceptions to all rules; and let him further bear in mind that the decision of the sex is not so much owing to the influence of the courses as the age of the ovum at the time it is fecundated, and indeed to more positively insure the production of a daughter, I would advise that coition be postponed till four or five days after the courses, and that it should not be repeated after the first half of the intermenstrual period; and to be most certain of producing a son, it should only be allowed during the latter half of the intermenstrual period. With these and the foregoing statements, it would seem superfluous for me further to state how physicians can prescribe to those who desire a boy, or a girl.

I am now prepared to reconcile in part the opinions of several authors, whose names I have mentioned, with my own views.

1st. As to Emerson's views and statistics showing that country women give birth to a greater proportion of boys than city women. The women of rural districts are more active and exercise more on foot than do city women; thus according to a previous statement of mine, they are more likely to have boys, for the following reasons: The ovum, if it has been matured a considerable time, will become a female if impregnated, and it makes a fastening within the uterus; but such an ovum is fecundated while within the uterus, or very nearly within it; now such an ovum thus far advanced is very likely to be cast off by the conquassatory movements the uterus undergoes in a rural woman while romping, running or lifting; while city women are more sedentary in their habits and employments, and thus such an ovum is more likely to make an undisturbed fastening; but if the ovum of the active exercising rural woman is fecundated while it is quite recent, it must take place while in the ovarium, or while in the first stage of its progress through the fallopian tube, and by the time it has reached the uterus it is ready at once to make a fastening, and is not so likely to be expelled as one fecundated in the uterus. Emerson further adduces statistics as previously mentioned, showing that during cholera seasons more females are conceived than males; this may be accounted for from the fact that at such times business is suspended to a great degree, and quietude is enjoined on all, and the women exercise less, and thus, in the manner I have explained, more females are conceived. These explanations will also apply to my observations and remarks that vigorous and active women average more boys than sickly, or delicate, or sedentary women, which observation is contrary to the opinion of many, that the strongest party regulates the sex.

As for the opinion of some statisticians, that the ratio of female births is greater among those born illegitimately than those born in wedlock, it may possibly be accounted for on the supposition that women are more susceptible of seduction shortly after than before their courses.

The ova of animals are matured at the time of rut, and at no other time can an animal be impregnated. The age of the ova corresponds to the length of time the animal has been in heat. Now as regards very young rams begetting more ewe lambs, while medium aged rams beget more males. I think it may possibly be accounted for by the fact that young rams cannot so readily distinguish a sheep in estuary from one that is not, and thus is more likely to neglect the former until the ovum has advanced to that condition in which it would grow to be a female, while the old ram immediately detects the sheep in heat, and thus the ovum is more likely to be impregnated as soon as it has become sufficiently matured.

The fact mentioned by Velpeau, that in a flock of sheep those that are first covered produce more females, may be accounted for from the fact that the ram does not enjoy his whole prolific energy until after a certain number of copulations, and is more likely to neglect the sheep in heat till the ova become old, while those that come immediately after them produce more males, because the ram has acquired more experience and prolific energy, and thus is more likely to cover them in the first stage of heat while the ova are recent, and afterward he becomes exhausted, gradually losing his strength, thus oftener neglecting the sheep as they first become in heat, until the ova become old, and consequently a greater number of ewes are produced.

According to my theories and explanations above given, the reasons are obvious why pigeons, doves, partridges, and many other birds that unite in couples during each season of their loves, produce nearly the same number of males as of females; while the gallinaceæ, the common fowl, on the contrary, and geese, ducks, turkeys, &c., where the same male suffices for several females, furnish many more females than males of their respective species; while bitches, cats and she wolves, which ordinarily permit the approaches of several dogs, &c., engender more males than females.

Those authors who have spoken of the fact that those animals which live in a state of "polygamy" produce many more females than males, have supposed that the same holds with the human species, as in Persia and Turkey; but it will be remembered that women admit of sexual intercourse at all times, consequently, according to my theories the chances of their producing boys and girls are about the same in a state of polygamy as in proper wedlock; but with animals, as I have already explained, it is very different.

M. Bailey and M. Villerme, and other authors who have made extensive researches to ascertain whether there is a greater ratio of girls born among the poor and wretched, and a greater proportion of boys among the affluent or comfortable, will see that, according to my theories and explanations, none of these opposite circumstances will affect the usual proportion of the sexes except as I have explained that physical inactivity and idleness favor the production of girls, while exercise and industry favor the production of boys.

If my views are correct, they overturn the opinion of those authors who have contended that it is probable that the nature of the sex is determined by that one of the couple whose prolific power, whether absolute or relative, is greatest at the moment of conception; and for like reasons disproportion of age of human parents will cause no material difference of the usual proportion of the sexes.

According to my theories I should expect that in placenta prævia cases the children are females, because, as I have said, the ovum which grows to be a female is probably fecundated while within the uterus; it therefore seems that it is more liable to form an attachmant to the neck of the uterus, than if it had been fecundated while within the ovary or fallopian tube. In this latter case I should expect that the semen had so impregnated it with growing and attaching powers, that it would fasten to the uterus as soon as it reached its cavity, and thus it would not be so liable to reach the cervix uteri as if it had been fecundated at a later period when it had advanced much nearer the cervix without attaching powers. However, not having had any placenta prævia cases, I may be mistaken, but at least it would do no hurt to note the sex of the child in all such cases.

And now having in view the good of mankind, I propose to all intelligent men to note all facts tending to prove or disprove the foregoing theories; and I also lay the following propositions before all intelligent agriculturists who raise stock:

1st. To test for male sheep; I propose that one or several middle aged healthy rams be confined to a small flock from the very beginning of the rutting season, or that several such divisions of a large flock be made. I should expect that a great share of new ova would be fecundated, and consequently that a great proportion of the lambs would be males.

2d. To test for female lambs: let one ram supply a large flock of sheep. I would expect that they would not all be served so readily in the beginning of heat as the small flock was, and thus a greater proportion of old ova would be impregnated, and consequently a greater number of the lambs would be females.

3d. To test for male colts, calves, pigs, dogs, &c., have the male admitted to the female as soon as she manifests the first indications of being in heat, and *vice versa;* to produce females let the female remain in heat several days before being admitted to the male.

I have accidentally witnessed some of the foregoing tests, and the results were always in favor of my theories; and I hope others will see proper to investigate the field which I have laid before them, and publish the results of their observations.

BUFFALO, March, 1855.

